# Comparative Clinical and Metabolic Outcomes of GLP-1 Receptor Agonists *Versus* SGLT2 Inhibitors in Type 1 Diabetes: A Retrospective Cohort Study from Saudi Arabia

**DOI:** 10.2174/0115733998428827251223081042

**Published:** 2026-02-13

**Authors:** Ayman Al Hayek, Asirvatham Alwin Robert, Abdullah Khalid Al Burayk, Abdulrhman Nasser Alwadai, Asma Mutni Almutairi, Abdullah Ali Shabani, Mohamed Abdulaziz Al Dawish

**Affiliations:** 1 Department of Endocrinology and Diabetes, Diabetes Treatment Center, Prince Sultan Military Medical City, Riyadh, Saudi Arabia

**Keywords:** Type 1 diabetes mellitus, GLP-1 receptor, SGLT2 inhibitors, glycemic control, sodium-glucose cotransporter-2, ischemic heart disease

## Abstract

**Introduction:**

This research is designed to compare the clinical characteristics, metabolic outcomes, and safety profiles of patients with Type 1 Diabetes (T1D) treated with Glucagon-Like Peptide-1 Receptor Agonists (GLP-1 RAs) against Sodium-Glucose co-transporter 2 (SGLT2) inhibitors in a real-world clinical setting over 12 months.

**Methods:**

Data from 112 adult T1D patients on intensive insulin therapy at Prince Sultan Military Medical City in Riyadh were examined for this retrospective cohort study. Among the 112 T1D, 96 were treated with GLP-1 receptor agonists and 16 were treated with SGLT2 inhibitors. Baseline demographics, the presence of comorbidities, diabetes complications, glycemic control (HbA1c), weight, insulin dosage, renal function, and lipid profiles were assessed at initiation and after 12 months of treatment.

**Results:**

Patients getting GLP-1 receptor agonists were younger (*p* = 0.006), had shorter diabetes duration (*p* = 0.030), and were more likely to use insulin pumps (*p* = 0.040). The SGLT2 group showed significantly higher albumin-to-creatinine ratios (*p* = 0.047) and higher statin use (*p* = 0.038). GLP-1 therapy led to significant HbA1c (*p* < 0.05) and weight reduction, while SGLT2 treatment improved renal markers and reduced insulin dose. LDL cholesterol decreased significantly in the GLP-1 group (*p* = 0.014), while the SGLT2 group showed more favorable changes in HDL and total cholesterol. Comorbidities and diabetes related complications were similar between groups.

**Discussion:**

GLP-1 RAs produced notable reductions in HbA1c levels and weight reduction, while SGLT2 inhibitors offered renal protection and decreased insulin needs. These real-world findings emphasize the value of tailoring adjunct therapies to each patient’s profile. Personalized treatment strategies are crucial for optimizing metabolic outcomes in T1D.

**Conclusion:**

Both GLP-1 RAs and SGLT2 inhibitors effectively improve glycemic control and cardiovascular risk markers in T1D, with distinct advantages in weight loss and renal function, respectively. These findings highlight the value of personalized adjunct therapy choices to optimize patient outcomes. Larger prospective studies are needed to confirm long-term benefits and safety.

## 1 INTRODUCTION

Globally, approximately 9.5 million individuals live with Type 1 Diabetes (T1D), with an estimated incidence of 14.1 cases per 100,000 person-years among those under 20 years of age [1, 2]. In Saudi Arabia, recent studies report an even higher incidence of 30.8 per 100,000 per year among children aged 0-14 years, and the national prevalence in those under 20 is projected to be about 35,000 cases, making it one of the highest rates in the Middle East [3]. Even though extensive research has been conducted, the factors driving this increasing trend remain unclear. As in many countries, the rapid upsurge in T1D cases in Saudi Arabia raises major concerns regarding disease control, management, and long-term health consequences, particularly among children and adolescents [4-8].

In recent years, attention has shifted toward using medications originally developed for type 2 diabetes in the management of T1D. Among these, glucagon-like peptide-1 receptor agonists (GLP-1 RAs) and sodium-glucose cotransporter-2 (SGLT2) inhibitors have developed as promising adjunct therapies and are sometimes prescribed off-label alongside insulin to advance treatment outcomes [9-12]. GLP-1 receptor agonists enhance glycemic control by stimulating glucose-dependent insulin, suppressing glucagon release, delaying gastric emptying, and promoting satiety, which together support weight reduction and improved glycemic control. Conversely, SGLT2 inhibitors reduce blood glucose levels by increasing urinary glucose excretion and offer additional benefits, such as decreased insulin demand and modest weight loss [13-15].

Although both medication classes have been studied in individuals with T1D, comparative evidence between GLP-1 receptor agonists and SGLT2 inhibitors is still limited, especially in real-world clinical settings [16]. Moreover, safety concerns, particularly the risk of diabetic ketoacidosis (DKA) associated with SGLT2 inhibitors, underscore the importance of cautious use. These factors highlight the need for further research to guide treatment decisions and determine which patient populations are most likely to benefit from these therapies [15, 17].

This retrospective cohort study aims to assess and compare the clinical characteristics, metabolic outcomes, and treatment responses of T1D patients treated with GLP-1 RAs versus SGLT2 inhibitors. By analyzing real-world patient data over a 12-month period, this study seeks to provide insight into the comparative effectiveness and safety of these two drug classes, supporting clinicians in tailoring adjunct therapy strategies to improve outcomes in individuals living with T1D.

## 2 METHODS

### 2.1 Study Design and Population

This retrospective observational study was conducted in September 2024 at the Diabetes Treatment Center, Prince Sultan Military Medical City (PSMMC), Riyadh. A retrospective chart review was performed for two time intervals: at baseline (prior to initiation of GLP-1RA/SGLT2 add-on therapy), during the first treatment year (January 2023), and during the second treatment year (January 2024). Safety outcomes, including hospitalizations due to DKA (including euglycemic DKA), severe hypoglycemia, and other treatment-related adverse events, were assessed through a manual chart review.

Participants were adults receiving intensive insulin therapy, either Multiple Daily Injections (MDI) or a conventional insulin pump, and attending regular follow-up visits at the diabetes center. A total of 112 patients were included in the analysis, with 96 in the GLP-1 RA group and 16 in the SGLT2 inhibitor group. Based on a post hoc power analysis (α = 0.05, power = 80%, medium effect size = 0.5), a minimum of 102 participants was required, and the study used a sample of 112 patients. The study was carried out in accordance with the Declaration of Helsinki and was approved by PSMMC’s Research and Ethics Committee (Approval No. HP-01-R092).

### 2.2 Inclusion and Exclusion Criteria

Eligible participants were identified from clinical records based on initiation of either GLP-1 RA or SGLT2 inhibitor therapy and follow-up for 12 months. Inclusion criteria included age ≥18 years, a confirmed diagnosis of T1D, and at least one year of follow-up data after starting treatment. Patients were excluded if they had incomplete data, follow-up of less than 12 months, concurrent enrollment in another investigational drug trial, pregnancy, chronic kidney disease (stage 4 or 5), or were on chronic dialysis, or had a history of concurrent steroid use.

### 2.3 Data Collection

Baseline characteristics, comorbidities, diabetes-related complications, and treatment-related clinical parameters were extracted from the electronic medical records of 1,440 patients with T1D. The variables collected included age, gender, weight, Body Mass Index (BMI), duration of diabetes, insulin therapy type (Multiple Daily Injections [MDI] *vs*. Insulin Pump Therapy [IPT]), statin use, and renal function indicators such as serum creatinine and urine Albumin-to-Creatinine Ratio (ACR).

Complications such as retinopathy, nephropathy, cerebrovascular accidents (CVA), ischemic heart disease (IHD), and peripheral vascular disease (PVD) were recorded. Comorbid autoimmune conditions, including celiac disease, hypothyroidism, and Graves’ disease, were also noted.

Laboratory values as well as glycated hemoglobin (HbA1c) and lipid profile (LDL, HDL, total cholesterol, and triglycerides) were obtained at baseline and at 12 months. Renal function indicators and insulin dose changes over the 12-month period were also measured.

### 2.4 Statistical Analysis

Data analysis was performed using Microsoft Excel 2019 (Microsoft Corporation, Redmond, WA, USA) and SPSS software version 22.0 (SPSS Inc., Chicago, IL, USA). Continuous variables were expressed as mean ± standard deviation (SD), while categorical variables were summarized as frequencies and percentages. Differences between the GLP-1 receptor agonist and SGLT2 inhibitor groups were evaluated using independent samples t-tests for continuous data and Fisher’s exact test for categorical variables. A p-value < 0.05 was considered indicative of statistical significance.

## 3 RESULTS

Table **1** summarizes the baseline characteristics of the study population, comparing patients treated with GLP-1 receptor agonists (n = 96) and those treated with SGLT2 inhibitors (n = 16). The mean age was significantly lower in the GLP-1 group compared to the SGLT2 group (31.1 ± 7.6 *vs*. 34.5 ± 10.8 years, *p* = 0.006). There were no significant differences in gender distribution between the two groups (GLP-1: 30 males/66 females; SGLT2: 6 males/10 females; *p* = 0.409). Although the GLP-1 group had a higher mean weight (93.1 ± 16.4 kg) and BMI (34.9 ± 5.11) compared to the SGLT2 group (80.1 ± 16.1 kg and 30.3 ± 6.4, respectively), these differences were not statistically significant (*p* = 0.651 and *p* = 0.216, respectively). The duration of diabetes was slightly longer in the SGLT2 group (16.5 ± 11.9 years) compared to the GLP-1 group (15.9 ± 8.17 years), with a statistically significant difference (*p* = 0.030).

Groups were compared using independent samples t-tests for continuous variables and Fisher’s exact test for categorical variables.

Table **2** summarizes the comparison of complications and comorbidities between the GLP-1 (n = 96) and SGLT2 (n = 16) groups. Celiac disease was reported in 16 patients in the GLP-1 group and none in the SGLT2 group, with a borderline statistical significance (*p* = 0.075). The prevalence of hypothyroidism was comparable between the groups (25% in GLP-1 *vs*. 25% in SGLT2; *p* = 0.626), as was the occurrence of Graves’ disease (3 in GLP-1, 0 in SGLT2; *p* = 0.625). Retinopathy was more common in the SGLT2 group (4 *vs*. 11 cases), but the difference was not statistically significant (*p* = 0.341). Nephropathy and peripheral vascular disease were rare; each was observed in only one patient in the GLP-1 group and none in the SGLT2 group (*p* = 0.857 for both). One case of ischemic heart disease (stent/CABG) was reported in the SGLT2 group, while none were reported in the GLP-1 group (*p* = 0.144). Cerebrovascular accidents were reported in one patient from each group, showing no significant difference (*p* = 0.266). Overall, no statistically significant differences in comorbidities or complications were observed between the two groups. Diabetic ketoacidosis was reported in 1 patient (6.3%) in the SGLT2 group, while no cases were observed in the GLP1 group.

Table **3** compares the clinical and treatment characteristics between the GLP-1 and SGLT2 groups. A significantly higher proportion of patients in the GLP-1 group were on Insulin Pump Therapy (IPT) compared to the SGLT2 group, where all patients were on Multiple Daily Injections (MDI) (*p* = 0.040). Statin use was significantly more frequent in the SGLT2 group (50%) than in the GLP-1 group (24%) (*p* = 0.038). The highest Albumin-to-Creatinine Ratio (ACR) was also significantly higher in the SGLT2 group (*p* = 0.047). Serum creatinine levels were comparable between the groups (63.5 ± 24.4 μmol/L in GLP-1 *vs*. 64.5 ± 22.7 μmol/L in SGLT2; *p* = 0.219).

Figure (**1**). illustrates that after 12 months, both the GLP-1 and SGLT2 treatment groups showed improvements in glycemic control, weight reduction, and insulin dose requirements compared to baseline. The mean HbA1c decreased from 8.8% to 8.2% (Δ = −0.6%) in the GLP1 group and from 9.3% to 8.7% (Δ = −0.6%) in the SGLT2 group, with a significant difference between groups at 12 months (*p* = 0.014). Mean body weight declined from 93.1 kg to 80.1 kg (Δ = −13.0 kg) in the GLP1 group and from 80.2 kg to 71.1 kg (Δ = −9.1 kg) in the SGLT2 group. The Total Daily Dose (TDD) of insulin decreased from 1.29 to 0.73 IU/kg/day (Δ = −0.56 IU/kg/day) in the GLP1 group and from 1.33 to 0.80 IU/kg/day (Δ = −0.53 IU/kg/day) in the SGLT2 group. Serum creatinine levels remained relatively stable over 12 months, with minor changes from 61.1 to 59.5 mmol/L (Δ = −1.6 mmol/L) in the GLP1 group and from 65.9 to 58.7 mmol/L (Δ = −7.2 mmol/L) in the SGLT2 group.

Figure (**2**) displays that after 12 months of treatment, both the GLP-1 and SGLT2 groups established favorable improvements in lipid parameters compared to baseline. The mean low-density lipoprotein (LDL) level decreased from 2.74 to 1.91 mmol/L (Δ = −0.83 mmol/L) in the GLP1 group and from 2.75 to 1.81 mmol/L (Δ = −0.94 mmol/L) in the SGLT2 group. Similarly, total cholesterol showed a reduction from 3.87 to 2.57 mmol/L (Δ = −1.30 mmol/L) in the GLP1 group and from 3.92 to 2.35 mmol/L (Δ = −1.57 mmol/L) in the SGLT2 group. In contrast, high-density lipoprotein (HDL) levels increased from 1.27 to 2.26 mmol/L (Δ = +0.99 mmol/L) in the GLP1 group and from 1.21 to 2.35 mmol/L (Δ = +1.14 mmol/L) in the SGLT2 group, indicating improvement in cardioprotective lipids. Triglyceride levels showed a modest decline over 12 months, from 1.65 to 1.36 mmol/L (Δ = −0.29 mmol/L) in the GLP1 group and from 1.42 to 1.26 mmol/L (Δ = −0.16 mmol/L) in the SGLT2 group. Overall, both therapies were associated with improvements in lipid profiles, with greater reductions in LDL and total cholesterol in the SGLT2 group.

## 4 DISCUSSION

This study provides a comparative analysis of clinical features, comorbid conditions, and treatment outcomes in patients with T1D receiving either GLP-1 receptor agonists or SGLT2 inhibitors. The results highlight both similarities and differences between the two drug classes in real-world settings. Notably, patients treated with GLP-1 receptor agonists were generally younger and had a shorter duration of diabetes, likely reflecting prescribing trends influenced by patient characteristics or clinician preferences. These findings are consistent with earlier studies suggesting that GLP-1 receptor agonists are often initiated earlier in the disease, predominantly in individuals [18, 19].

At baseline, weight and BMI were comparable between the two groups. Over 12 months, follow-up, treatment with GLP-1 receptor agonists was associated with greater weight loss, consistent with the well-established weight-reducing effects of this drug class [20]. Additionally, both GLP-1 RAs and SGLT2 inhibitors led to improvements in glycemic control, with GLP-1 therapy showing a more pronounced decline in HbA1c. These observations are in line with major trials, including ADJUNCT ONE and TWO, which reported dose-dependent falls in HbA1c, body weight, and insulin requirements with GLP-1 therapy, together with a higher incidence of mild hypoglycemia and ketosis [21-25]. SGLT2 inhibitors also demonstrated additional benefits, together with improved renal function and reduced albuminuria, as well as decreased total daily insulin requirements and enhanced glycemic control without increasing the risk of severe hypoglycemia [21-23, 26, 27]. Consistent with results from the EASE and DEPICT trials and related studies, exhibited that SGLT2 inhibitors enhanced glycemic control, decreased body weight, and lowered insulin requirements, but carried a slightly raised risk of diabetic ketoacidosis [28-31]. In addition, SGLT2 inhibitors provided additional benefits, including reduced insulin requirements and improvements in markers of renal function. These findings are consistent with evidence from randomized controlled trials demonstrating the kidney-protective effects of SGLT2 inhibitors, such as their ability to lower albuminuria, which may explain the higher baseline Albumin-to-Creatinine Ratio (ACR) in the SGLT2 group and the subsequent progress observed during follow-up [32, 33]. Overall, accumulating evidence indicates that SGLT2 inhibitors improve glycemic control, decrease total daily insulin needs, and promote weight loss in T1D patients without increasing the risk of severe hypoglycemia [15, 34-37].

With respect to cardiovascular risk factors, both treatment strategies were associated with favorable lipid profile changes. Patients treated with GLP-1 receptor agonists showed greater reductions in Low-Density Lipoprotein Cholesterol (LDL-C), whereas those getting SGLT2 inhibitors experienced more significant increases in High-Density Lipoprotein Cholesterol (HDL-C) along with reductions in total cholesterol. These dissimilar effects on lipid levels align with results from clinical trials and meta-analyses, which report that GLP-1 receptor agonists largely lower triglycerides and LDL-C, whereas SGLT2 inhibitors tend to produce modest increases in HDL-C [38, 39].

In this study, the prevalence of comorbid conditions, including hypothyroidism and Graves’ disease, was similar between the two treatment groups. Although celiac disease was more frequently observed among patients receiving GLP-1 receptor agonists, the difference was not statistically significant. The overall low incidence of complications such as nephropathy, ischemic heart disease, and cerebrovascular events may reflect the younger age of the cohort and their management at a specialized diabetes center. Notably, insulin pump use was substantially higher in the GLP-1 receptor agonist group, which suggests either a more complex baseline therapy or a more intensive approach to glucose control. In contrast, all patients treated with SGLT2 inhibitors relied on multiple daily injections, likely due to standard clinical practice patterns or differences in resource availability [40, 41]. Although both GLP-1 receptor agonists and SGLT2 inhibitors show promising benefits as adjunctive therapies for T1D, their safe integration in routine care requires careful patient selection, continuous monitoring, and further long-term research [42].

## CONCLUSION

This study demonstrates that both GLP-1 receptor agonists and SGLT2 inhibitors effectively improve glycemic control and cardiovascular risk factors in individuals with type 1 diabetes. GLP-1 RAs were associated with greater reductions in body weight and HbA1c reduction, whereas SGLT2 inhibitors showed advantages in insulin dose decrease and improvements in renal function. Lipid profile changes differ between groups, reflecting distinct therapeutic effects. Comorbidity profiles were similar, although insulin pump use was more common among patients receiving GLP-1 RAs. Despite the smaller sample size in the SGLT2 group, the findings underscore the complementary roles of these agents in tailored diabetes management. Future prospective studies with larger cohorts and longer follow-up are necessary to further elucidate the long-term comparative effectiveness and safety profiles of these agents.

## LIMITATIONS

The present study has few limitations. The relatively small sample size, retrospective nature, and single-center setting may limit the generalizability of the findings. Moreover, the marked difference in group sizes (GLP-1, n = 96; SGLT2, n = 16) may have influenced the statistical power. Certain potential confounders, including treatment duration, comorbid conditions, and patient adherence, were not completely accounted for. Therefore, future prospective and multicenter research with larger, more balanced cohorts is recommended to confirm and expand upon these findings.

## Figures and Tables

**Fig. (1):**
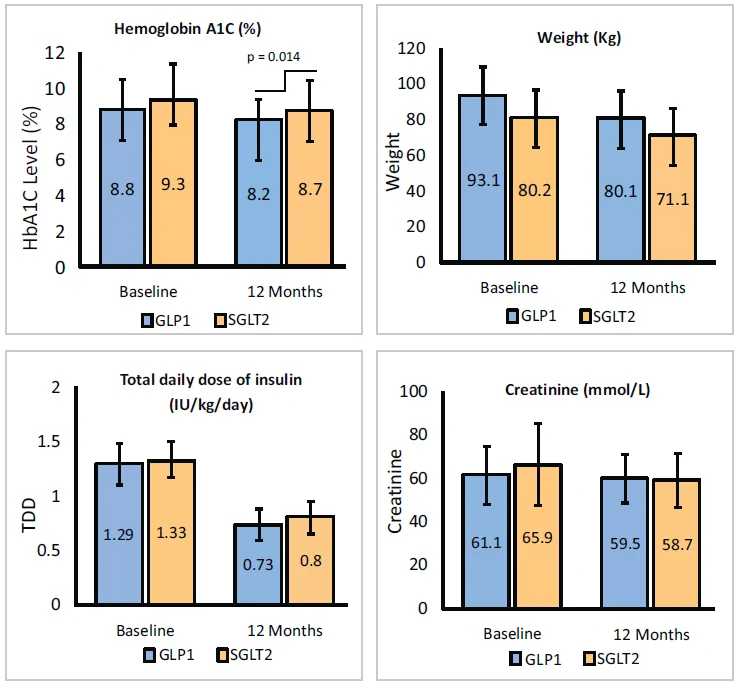
Change in outcome over 12 months of GLP1 and SGLT2 therapy groups. Clinical outcomes (HbA1c, weight, insulin dose, and creatinine) were compared between GLP-1 and SGLT2 groups using independent t-tests, with significance set at *p* < 0.05. Glycated Hemoglobin (HbA1c); Total daily dose (TDD); Glucagon-Like Peptide-1 Receptor Agonist (GLP-1); Sodium-Glucose Co-Transporter 2 (SGLT2).

**Fig. (2):**
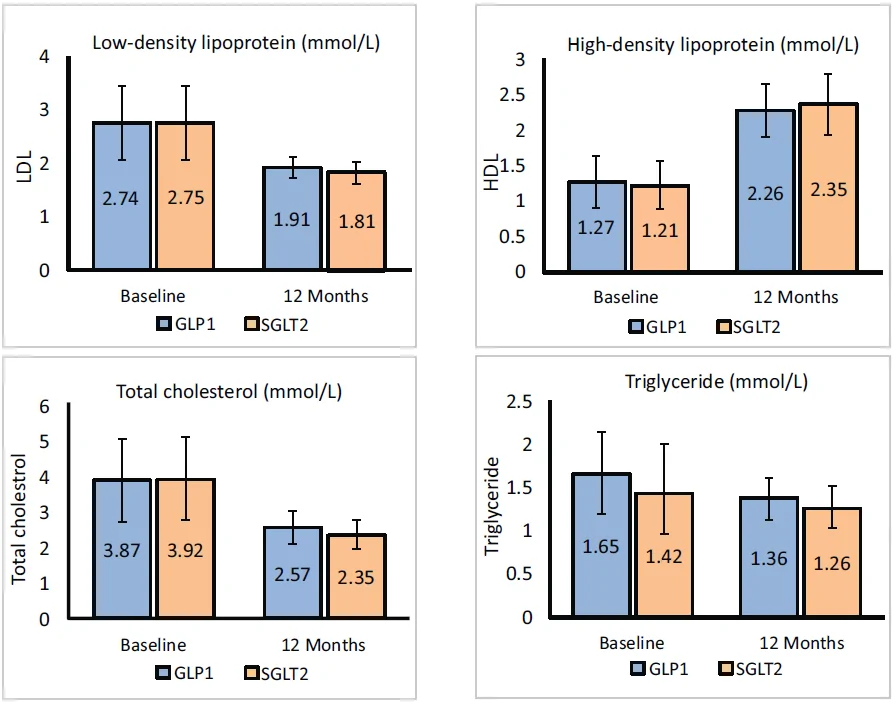
Change in lipid profile over 12 months of GLP1 and SGLT2 therapy groups. Lipid parameters (LDL, HDL, total cholesterol, and triglycerides) were compared between GLP-1 and SGLT2 groups using independent t-tests, with significance set at *p* < 0.05. Low-Density Lipoprotein (LDL); High-Density Lipoprotein (HDL); Glucagon-Like Peptide-1 Receptor Agonist (GLP-1); Sodium-Glucose Co-Transporter 2 (SGLT2).

**Table 1:** Baseline Characteristics of the study population.

**Variables**	**GLP1 (n,96)**	**SGLT2 (n, 16)**	***p* value**
Age (mean ± SD)	31.1 ± 7.6	34.5 ± 10.8	0.006
Gender (Male/Female)	30/66	6/10	0.409
Weight	93.1 ± 16.4	80.1 ± 16.1	0.651
Body mass index	34. 9 ± 5.11	30.3 ± 6.4	0.216
Duration of diabetes	15. 9 ± 8.17	16.5 ± 11.9	**0.030**

**Table 2:** Comparison of complications and comorbidities between GLP-1 and SGLT2 groups.

**Complications and Comorbidities**	**GLP1 (n, 96)**	**SGLT2 (n, 16)**	***p* value**
Celiac disease	16	0	0.075
Hypothyroidism	24	4	0.626
Graves’ disease	3	0	0.625
Retinopathy	11	4	0.341
Nephropathy	1	0	0.857
Ischemic Heart Disease (Stent/CABG)	0	1	0.144
Peripheral Vascular Disease	1	0	0.857
Cerebrovascular Accident	1	1	0.266

Groups compared by Fisher’s exact test.

**Table 3:** Comparison of clinical and treatment characteristics between GLP1 and SGLT2 therapy groups.

**Variables**	**GLP1 (n,96)**	**SGLT2 (n, 16)**	***p* value**
MDI/IPT	77/19	16/0	**0.040**
Statin therapy	23	8	**0.038**
Highest Albumin-to-Creatinine Ratio (ACR)	10	5	**0.047**
Creatinine (μmol/L)	63.5 ± 24.4	64.5 ± 22.7	0.219

Groups compared by Fisher’s exact test, MDI: Multiple Daily Injection of Insulin; IPT: Insulin Pump Therapy.

## Data Availability

The data and supportive information are available within the article.

## LIST OF ABBREVIATIONS

ACR: Albumin-to-Creatinine Ratio
BMI: Body Mass Index
CVA: Cerebrovascular Accident
DKA: Diabetic Ketoacidosis
GLP-1 RA: Glucagon-Like Peptide-1 Receptor Agonist
HbA1c: Glycated Hemoglobin
HDL: High-Density Lipoprotein
IHD: Ischemic Heart Disease
IPT: Insulin Pump Therapy
LDL: Low-Density Lipoprotein
MDI: Multiple Daily Injections
PVD: Peripheral Vascular Disease
SGLT2: Sodium-Glucose Co-Transporter 2
T1D: Type 1 Diabetes
T2DM: Type 2 Diabetes Mellitus
TDD: Total Daily Dose

## STANDARDS OF REPORTING

STROBE guidelines were followed.
